# Supplementary material for: A systemic approach to estimate and validate RP-HPLC assay method for remdesivir and favipiravir in capsule dosage form
Source: PLoS One. 2025 Apr 15;20(4):e0321474. doi: 10.1371/journal.pone.0321474 (PMC11999136; doi:10.1371/journal.pone.0321474)
Supplement: S1 Table — (DOCX) [file pone.0321474.s001.docx]

**Table S1: Solution Stability Favipiravir**

| **Areas** | **Average Area** | **% Recovery** | **STDEV** | **% RSD** | **% Diff.** | **Parameters** |
| --- | --- | --- | --- | --- | --- | --- |
| 918903.55 | 918636.2633 | - | 1811.874 | 0.198% | - | std |
| 917490.74 |  |  |  |  |  |  |
| 915307.23 |  |  |  |  |  |  |
| 923897.89 |  |  |  |  |  |  |
| 917581.91 |  |  |  |  |  |  |
| 916942.49 | 917577.78 | 99.88% | 4360.3168 | 0.475% | 0.11% | Initial |
| 913569.96 |  |  |  |  |  |  |
| 922220.89 |  |  |  |  |  |  |
| 901243.20 | 896850.90 | 97.63% | 5430.492 | 0.606% | 2.31% | 25C-24H |
| 890779.05 |  |  |  |  |  |  |
| 898530.44 |  |  |  |  |  |  |
| 911933.54 | 911117.0303 | 99.18% | 943.035 | 0.104% | 0.70% | 4C-24H |
| 910084.84 |  |  |  |  |  |  |
| 911332.71 |  |  |  |  |  |  |
| 862490.31 | 866736.0372 | 94.35% | 4005.508 | 0.462% | 5.86% | 25C-48H |
| 870447.75 |  |  |  |  |  |  |
| 867270.05 |  |  |  |  |  |  |
| 884351.31 | 888793.2687 | 96.75% | 5316.445 | 0.598% | 3.23% | 4C-48H |
| 894683.90 |  |  |  |  |  |  |
| 887344.60 |  |  |  |  |  |  |
| 799531.16 | 797122.2707 | 86.77% | 6833.514 | 0.857% | 15.11% | 25C-72H |
| 802425.12 |  |  |  |  |  |  |
| 789410.53 |  |  |  |  |  |  |
| 870562.78 | 861300.4300 | 93.76% | 8022.969 | 0.931% | 6.53% | 4C-72H |
| 856512.03 |  |  |  |  |  |  |
| 856826.48 |  |  |  |  |  |  |
