# Supplementary material for: A systemic approach to estimate and validate RP-HPLC assay method for remdesivir and favipiravir in capsule dosage form
Source: PLoS One. 2025 Apr 15;20(4):e0321474. doi: 10.1371/journal.pone.0321474 (PMC11999136; doi:10.1371/journal.pone.0321474)
Supplement: S2 Table — (DOCX) [file pone.0321474.s002.docx]

**Table S2: Solution Stability Remdesivir**

| **Areas** | **Average Area** | **% Recovery** | **STDEV** | **% RSD** | **% Diff.** | **Parameters** |
| --- | --- | --- | --- | --- | --- | --- |
| 124218.47 | 124509.5279 | - | 364.885 | 0.293% | - | std |
| 124869.66 |  |  |  |  |  |  |
| 124829.36 |  |  |  |  |  |  |
| 124228.28 |  |  |  |  |  |  |
| 124401.88 |  |  |  |  |  |  |
| 124126.63 | 124203.09 | 99.75% | 75.0852 | 0.060% | 0.24% | Initial |
| 124276.72 |  |  |  |  |  |  |
| 124205.91 |  |  |  |  |  |  |
| 121104.42 | 120731.24 | 96.97% | 520.860 | 0.431% | 3.04% | 25C-24H |
| 120953.12 |  |  |  |  |  |  |
| 120136.18 |  |  |  |  |  |  |
| 123117.68 | 123182.2879 | 98.93% | 557.136 | 0.452% | 1.06% | 4C-24H |
| 123768.91 |  |  |  |  |  |  |
| 122660.27 |  |  |  |  |  |  |
| 116449.36 | 116408.5630 | 93.49% | 176.970 | 0.152% | 6.52% | 25C-48H |
| 116214.76 |  |  |  |  |  |  |
| 116561.57 |  |  |  |  |  |  |
| 118849.14 | 119470.9003 | 95.95% | 543.729 | 0.455% | 4.05% | 4C-48H |
| 119857.32 |  |  |  |  |  |  |
| 119706.23 |  |  |  |  |  |  |
| 105086.50 | 104225.0944 | 83.71% | 770.966 | 0.740% | 16.33% | 25C-72H |
| 103599.78 |  |  |  |  |  |  |
| 103989.01 |  |  |  |  |  |  |
| 116156.97 | 115256.0077 | 92.57% | 782.499 | 0.679% | 7.45% | 4C-72H |
| 114864.67 |  |  |  |  |  |  |
| 114746.38 |  |  |  |  |  |  |
