# Supplementary material for: A systemic approach to estimate and validate RP-HPLC assay method for remdesivir and favipiravir in capsule dosage form
Source: PLoS One. 2025 Apr 15;20(4):e0321474. doi: 10.1371/journal.pone.0321474 (PMC11999136; doi:10.1371/journal.pone.0321474)
Supplement: S3 Table — (DOCX) [file pone.0321474.s003.docx]

**Table S3: Linearity Test**

| **Linearity (Favipiravir)** | | | | | |
| --- | --- | --- | --- | --- | --- |
|  | | | | | |
| **Parameters** | **50%** | **75%** | **100%** | **125%** | **150%** |
| **Conc. (mg/ml)** | 0.04 | 0.06 | 0.08 | 0.10 | 0.12 |
| **Injection # 1** | 420754.00 | 638769.67 | 850309.52 | 1071693.75 | 1282417.27 |
| **Injection # 2** | 420512.21 | 636604.63 | 854492.61 | 1067284.01 | 1281435.14 |
| **Injection # 3** | 422020.31 | 642730.09 | 850940.72 | 1069466.04 | 1285751.01 |
| **Average Area** | 421095.51 | 639368.13 | 851914.28 | 1069481.27 | 1283201.14 |
| **Slope** | 10771622.00 | | | | |
| **Intercept** | 206714.75 | | | | |
| **Correlation** | 1.000 | | | | |
|  |  |  |  |  |  |
|  |  |  |  |  |  |
| **Linearity (Remdesivir)** | | | | | |
|  | | | | | |
| **Parameters** | **50%** | **75%** | **100%** | **125%** | **150%** |
| **Conc. (mg/ml)** | 0.02 | 0.03 | 0.04 | 0.05 | 0.06 |
| **Injection # 1** | 59420.66 | 89987.60 | 119936.10 | 150246.91 | 180740.29 |
| **Injection # 2** | 60399.32 | 89953.73 | 119921.91 | 150571.05 | 180400.71 |
| **Injection # 3** | 60124.30 | 90344.62 | 120127.73 | 150683.96 | 181110.57 |
| **Average Area** | 59981.43 | 90095.32 | 119995.25 | 150500.64 | 180750.53 |
| **Slope** | 1509717.59 | | | | |
| **Intercept** | 29681.58 | | | | |
| **Correlation** | 1.000 | | | | |
