# Supplementary material for: A systemic approach to estimate and validate RP-HPLC assay method for remdesivir and favipiravir in capsule dosage form
Source: PLoS One. 2025 Apr 15;20(4):e0321474. doi: 10.1371/journal.pone.0321474 (PMC11999136; doi:10.1371/journal.pone.0321474)
Supplement: S4 Table — (DOCX) [file pone.0321474.s004.docx]

**Table S4: Precision Favipiravir Analyst 01**

| Areas | Results | Average | SD | STDEV |
| --- | --- | --- | --- | --- |
| 918233 |  |  |  |  |
| 912956 |  |  |  |  |
| 913434 |  | - |  |  |
| 919593 |  |  |  |  |
| 913434 |  |  |  |  |
| 920417 | 99.47 | 915954.92 | 3955.247 | 0.432% |
| 912211 | 100.36 |  |  |  |
| 917036 | 99.84 |  |  |  |
| 920555 | 99.45 |  |  |  |
| 913615 | 100.21 |  |  |  |
| 911895 | 100.40 |  |  |  |
