# Supplementary material for: A systemic approach to estimate and validate RP-HPLC assay method for remdesivir and favipiravir in capsule dosage form
Source: PLoS One. 2025 Apr 15;20(4):e0321474. doi: 10.1371/journal.pone.0321474 (PMC11999136; doi:10.1371/journal.pone.0321474)
Supplement: S5 Table — (DOCX) [file pone.0321474.s005.docx]

**Table S5: Precision Remdesivir Analyst 01**

| Areas | Results | Average | SD | STDEV |
| --- | --- | --- | --- | --- |
| 122665 |  |  |  |  |
| 121792 |  |  |  |  |
| 122505 |  | - |  |  |
| 122957 |  |  |  |  |
| 122505 |  |  |  |  |
| 122702 | 99.83 | 122363.34 | 511.691 | 0.418% |
| 121788 | 100.58 |  |  |  |
| 122082 | 100.33 |  |  |  |
| 122944 | 99.63 |  |  |  |
| 122804 | 99.74 |  |  |  |
| 121860 | 100.52 |  |  |  |
