# Supplementary material for: A systemic approach to estimate and validate RP-HPLC assay method for remdesivir and favipiravir in capsule dosage form
Source: PLoS One. 2025 Apr 15;20(4):e0321474. doi: 10.1371/journal.pone.0321474 (PMC11999136; doi:10.1371/journal.pone.0321474)
Supplement: S6 Table — (DOCX) [file pone.0321474.s006.docx]

**Table S6: Precision Favipiravir Analyst 02**

| Areas | Results | Average | SD | STDEV |
| --- | --- | --- | --- | --- |
| 930363 |  |  |  |  |
| 930786 |  |  | - |  |
| 925736 |  |  |  |  |
| 929223 |  |  |  |  |
| 925736 |  |  |  |  |
| 928499 | 99.99 | 928454.62 | 2580.431 | 0.278% |
| 926995 | 100.15 |  |  |  |
| 931548 | 99.66 |  |  |  |
| 931555 | 99.66 |  |  |  |
| 925536 | 100.31 |  |  |  |
| 926595 | 100.19 |  |  |  |
