# Supplementary material for: A systemic approach to estimate and validate RP-HPLC assay method for remdesivir and favipiravir in capsule dosage form
Source: PLoS One. 2025 Apr 15;20(4):e0321474. doi: 10.1371/journal.pone.0321474 (PMC11999136; doi:10.1371/journal.pone.0321474)
Supplement: S7 Table — (DOCX) [file pone.0321474.s007.docx]

**Table S7: Precision Remdesivir Analyst 02**

| Areas | Results | Average | SD | STDEV |
| --- | --- | --- | --- | --- |
| 121420 |  |  |  |  |
| 121232 |  |  |  |  |
| 121575 |  |  | - |  |
| 121177 |  |  |  |  |
| 121575 |  |  |  |  |
| 120518 | 100.73 | 120848.47 | 331.924 | 0.275% |
| 120887 | 100.42 |  |  |  |
| 121398 | 100.00 |  |  |  |
| 120802 | 100.49 |  |  |  |
| 120504 | 100.74 |  |  |  |
| 120981 | 100.34 |  |  |  |
