# Supplementary material for: A systemic approach to estimate and validate RP-HPLC assay method for remdesivir and favipiravir in capsule dosage form
Source: PLoS One. 2025 Apr 15;20(4):e0321474. doi: 10.1371/journal.pone.0321474 (PMC11999136; doi:10.1371/journal.pone.0321474)
Supplement: S8 Table — (DOCX) [file pone.0321474.s008.docx]

**Table S8: Accuracy Favipiravir**

| **Area** | **% Assay** | **% Recovered** | **% RSD** | **Mean Recovery** | |
| --- | --- | --- | --- | --- | --- |
| 893114 | - | - | - | - | |
| 896194 |  |  |  |  |  |
| 892326 |  |  |  |  |  |
| 890638 |  |  |  |  |  |
| 892326 |  |  |  |  |  |
| 531057 | 59.41% | 99.02% | 0.055% | 98.95% | |
| 530552 | 59.35% | 98.92% |  |  |  |
| 530552 | 59.35% | 98.92% |  |  |  |
| 709014 | 79.32% | 99.15% | 0.349% | 99.54% | |
| 713824 | 79.86% | 99.82% |  |  |  |
| 712521 | 79.71% | 99.64% |  |  |  |
| 893483 | 99.96% | 99.96% | 0.288% | 100.00% | |
| 896645 | 100.31% | 100.31% |  |  |  |
| 891539 | 99.74% | 99.74% |  |  |  |
| 1076826 | 120.47% | 100.39% | 0.139% | 100.24% | |
| 1075179 | 120.28% | 100.24% |  |  |  |
| 1073834 | 120.13% | 100.11% |  |  |  |
| 1256000 | 140.51% | 100.37% | 0.313% | 100.05% | |
| 1248164 | 139.63% | 99.74% |  |  |  |
| 1252185 | 140.08% | 100.06% |  |  |  |
| Minimum % Recovery = | | | | | 98.95% |
| Maximum % Recovery = | | | | | 100.24% |
| Mean % Recovery = | | | | | 99.76% |
