# Supplementary material for: A systemic approach to estimate and validate RP-HPLC assay method for remdesivir and favipiravir in capsule dosage form
Source: PLoS One. 2025 Apr 15;20(4):e0321474. doi: 10.1371/journal.pone.0321474 (PMC11999136; doi:10.1371/journal.pone.0321474)
Supplement: S9 Table — (DOCX) [file pone.0321474.s009.docx]

**Table S9: Accuracy Remdesivir**

| **Area** | **% Assay** | **% Recovered** | **% RSD** | **Mean Recovery** | |
| --- | --- | --- | --- | --- | --- |
| 126270 | - | - | - | - | |
| 126381 |  |  |  |  |  |
| 126048 |  |  |  |  |  |
| 126323 |  |  |  |  |  |
| 126048 |  |  |  |  |  |
| 75228 | 59.60% | 99.34% | 0.067% | 99.26% | |
| 75141 | 59.53% | 99.22% |  |  |  |
| 75141 | 59.53% | 99.22% |  |  |  |
| 101077 | 80.08% | 100.11% | 0.359% | 100.14% | |
| 100769 | 79.84% | 99.80% |  |  |  |
| 101493 | 80.41% | 100.52% |  |  |  |
| 126143 | 99.94% | 99.94% | 0.268% | 100.06% | |
| 126061 | 99.88% | 99.88% |  |  |  |
| 126683 | 100.37% | 100.37% |  |  |  |
| 151473 | 120.01% | 100.01% | 0.266% | 100.15% | |
| 151421 | 119.97% | 99.98% |  |  |  |
| 152144 | 120.54% | 100.45% |  |  |  |
| 176740 | 140.03% | 100.02% | 0.099% | 99.98% | |
| 176796 | 140.08% | 100.05% |  |  |  |
| 176468 | 139.82% | 99.87% |  |  |  |
| Minimum % Recovery = | | | | | 99.26% |
| Maximum % Recovery = | | | | | 100.15% |
| Mean % Recovery = | | | | | 99.92% |
