# Supplementary material for: A systemic approach to estimate and validate RP-HPLC assay method for remdesivir and favipiravir in capsule dosage form
Source: PLoS One. 2025 Apr 15;20(4):e0321474. doi: 10.1371/journal.pone.0321474 (PMC11999136; doi:10.1371/journal.pone.0321474)
Supplement: S10 Table — (DOCX) [file pone.0321474.s010.docx]

**Table S10: Robustness Favipiravir**

| **Areas** | **Average** | **Results** | **SD** | **% RSD** | **% Dev** | **Parameter** |
| --- | --- | --- | --- | --- | --- | --- |
| 937734.36 | 935092.7373 |  | 2271.755 | 0.243% | - | std |
| 934389.71 |  |  |  |  |  |  |
| 933398.84 |  | - |  |  |  |  |
| 936541.93 |  |  |  |  |  |  |
| 933398.84 |  |  |  |  |  |  |
| 1025334.67 | 1027892.92 | 110.16% | 3876.058 | 0.377% | -9.221% | +0.2mL |
| 1032352.50 |  |  |  |  |  |  |
| 1025991.57 |  |  |  |  |  |  |
| 1394255.97 | 1395006.16 | 150.18% | 2652.644 | 0.190% | -33.413% | -0.2mL |
| 1397953.11 |  |  |  |  |  |  |
| 1392809.41 |  |  |  |  |  |  |
| 797886.50 | 794940.2476 | 85.22% | 4816.954 | 0.606% | 17.342% | 3nm |
| 797552.80 |  |  |  |  |  |  |
| 789381.45 |  |  |  |  |  |  |
| 745462.17 | 748157.0641 | 80.10% | 2345.418 | 0.313% | 24.847% | -3nm |
| 749737.20 |  |  |  |  |  |  |
| 749271.83 |  |  |  |  |  |  |
| 831214.15 | 833282.46 | 88.57% | 4846.52 | 0.581% | 12.904% | +(5%) |
| 838819.98 |  |  |  |  |  |  |
| 829813.24 |  |  |  |  |  |  |
| 865617.78 | 862942.91 | 92.28% | 2316.516 | 0.268% | 8.367% | -(5%) |
| 861611.60 |  |  |  |  |  |  |
| 861599.34 |  |  |  |  |  |  |
| 931554.57 |  |  |  |  |  | Column 4µm C18, 150mm change |
| 931074.43 | 862942.91 | 99.52% | 1431.901 | 0.153% | 0.48% |  |
| 928869.48 |  |  |  |  |  |  |
