# Supplementary material for: A systemic approach to estimate and validate RP-HPLC assay method for remdesivir and favipiravir in capsule dosage form
Source: PLoS One. 2025 Apr 15;20(4):e0321474. doi: 10.1371/journal.pone.0321474 (PMC11999136; doi:10.1371/journal.pone.0321474)
Supplement: S11 Table — (DOCX) [file pone.0321474.s011.docx]

**Table S11: Robustness Remdesivir**

|  |  |  | |  | |  | |  | |  |  |  |
| --- | --- | --- | --- | --- | --- | --- | --- | --- | --- | --- | --- | --- |
| **Areas** | **Average** | | **Results** | | **SD** | | **% RSD** | | **% Dev** | | **Parameter** |  |
| 124852.76 | 124240.6315 | |  | | 481.274 | | 0.387% | | - | | std |  |
| 124468.68 |  |  |  | |  |  |  |  |  |  |  |  |
| 123896.37 |  |  |  | |  |  |  |  |  |  |  |  |
| 124088.97 |  |  |  | |  |  |  |  |  |  |  |  |
| 123896.37 |  |  |  | |  |  |  |  |  |  |  |  |
| 137175.28 | 137144.41 | | 110.36% | | 197.184 | | 0.144% | | -9.385% | | +0.2mL |  |
| 137324.34 |  |  |  |  |  |  |  |  |  |  |  |  |
| 136933.61 |  |  |  |  |  |  |  |  |  |  |  |  |
| 130213.27 | 130761.01 | | 105.32% | | 481.441 | | 0.368% | | -5.055% | | -0.2mL |  |
| 131117.14 |  |  |  |  |  |  |  |  |  |  |  |  |
| 130952.62 |  |  |  |  |  |  |  |  |  |  |  |  |
| 105546.23 | 105515.8179 | | 85.40% | | 334.642 | | 0.317% | | 17.093% | | 3nm |  |
| 105167.01 |  |  |  |  |  |  |  |  |  |  |  |  |
| 105834.22 |  |  |  |  |  |  |  |  |  |  |  |  |
| 99065.92 | 99206.8670 | | 80.13% | | 396.240 | | 0.399% | | 24.805% | | -3nm |  |
| 98900.37 |  |  |  |  |  |  |  |  |  |  |  |  |
| 99654.31 |  |  |  |  |  |  |  |  |  |  |  |  |
| 110316.59 | 110546.34 | | 88.36% | | 878.294 | | 0.794% | | 13.167% | | +(5%) |  |
| 111516.68 |  |  |  |  |  |  |  |  |  |  |  |  |
| 109805.76 |  |  |  |  |  |  |  |  |  |  |  |  |
| 115315.09 | 115792.61 | | 92.19% | | 486.5588 | | 0.420% | | 8.474% | | -(5%) |  |
| 116287.73 |  |  |  |  |  |  |  |  |  |  |  |  |
| 115775.01 |  |  |  |  |  |  |  |  |  |  |  |  |
| 123816.55 | 123257.74 | | 99.41% | | 484.0071 | | 0.392% | | 0.51% | | Column 4µm C18, 150mm change |  |
| 122963.59 |  |  |  |  |  |  |  |  |  |  |  |  |
| 122993.67 |  |  |  |  |  |  |  |  |  |  |  |  |
