# Supplementary material for: A systemic approach to estimate and validate RP-HPLC assay method for remdesivir and favipiravir in capsule dosage form
Source: PLoS One. 2025 Apr 15;20(4):e0321474. doi: 10.1371/journal.pone.0321474 (PMC11999136; doi:10.1371/journal.pone.0321474)
Supplement: S12 Table — (DOCX) [file pone.0321474.s012.docx]

**Table S12: Stress Degradation Favipiravir**

| **Areas** | **Average Area** | **% Recovery** | **STDEV** | **% RSD** | **% Diff.** | **Parameters** |
| --- | --- | --- | --- | --- | --- | --- |
| 916412.70 | 916232.1375 | - | 611.697 | 0.067% | - | std |
| 915767.73 |  |  |  |  |  |  |
| 915189.92 |  |  |  |  |  |  |
| 916496.40 |  |  |  |  |  |  |
| 917293.93 |  |  |  |  |  |  |
| 736461.28 | 741139.92 | 80.89% | 5892.4423 | 0.795% | 23.625% | 70 C |
| 747757.51 |  |  |  |  |  |  |
| 739200.97 |  |  |  |  |  |  |
| 827243.68 | 825283.93 | 90.07% | 5684.444 | 0.689% | 11.020% | 40 C & RH 75% |
| 829729.22 |  |  |  |  |  |  |
| 818878.89 |  |  |  |  |  |  |
| 782956.25 | 779349.1106 | 85.06% | 3183.497 | 0.408% | 17.564% | Acid Treatment |
| 778158.78 |  |  |  |  |  |  |
| 776932.30 |  |  |  |  |  |  |
| 809675.70 | 809251.6712 | 88.32% | 5320.757 | 0.657% | 13.220% | Base Treatment |
| 803731.59 |  |  |  |  |  |  |
| 814347.73 |  |  |  |  |  |  |
| 863245.59 | 861012.6934 | 93.97% | 2221.555 | 0.258% | 6.824% | Peroxide Treatment |
| 860989.83 |  |  |  |  |  |  |
| 858802.66 |  |  |  |  |  |  |
| 888106.00 | 883462.4199 | 96.42% | 5980.419 | 0.677% | 4.049% | 1.2 M Lux |
| 876714.20 |  |  |  |  |  |  |
| 885567.06 |  |  |  |  |  |  |
