# Supplementary material for: A systemic approach to estimate and validate RP-HPLC assay method for remdesivir and favipiravir in capsule dosage form
Source: PLoS One. 2025 Apr 15;20(4):e0321474. doi: 10.1371/journal.pone.0321474 (PMC11999136; doi:10.1371/journal.pone.0321474)
Supplement: S13 Table — (DOCX) [file pone.0321474.s013.docx]

**Table S13: Stress Degradation Remdesivir**

| **Areas** | **Average Area** | **% Recovery** | **STDEV** | **% RSD** | **% Diff.** | **Parameters** |
| --- | --- | --- | --- | --- | --- | --- |
| 122907.99 | 123066.8385 | - | 288.271 | 0.235% | - | std |
| 123189.69 |  |  |  |  |  |  |
| 122613.20 |  |  |  |  |  |  |
| 123257.17 |  |  |  |  |  |  |
| 123366.13 |  |  |  |  |  |  |
| 98106.75 | 99541.00 | 80.88% | 1328.795 | 1.335% | 23.634% | 70 C |
| 99786.01 |  |  |  |  |  |  |
| 100730.24 |  |  |  |  |  |  |
| 109983.77 | 110099.81 | 89.46% | 1280.903 | 1.163% | 11.778% | 40 C & RH 75% |
| 111434.78 |  |  |  |  |  |  |
| 108880.87 |  |  |  |  |  |  |
| 104262.21 | 104374.7338 | 84.81% | 552.180 | 0.529% | 17.909% | Acid Treatment |
| 104974.51 |  |  |  |  |  |  |
| 103887.48 |  |  |  |  |  |  |
| 108611.73 | 108744.1737 | 88.36% | 973.123 | 0.895% | 13.171% | Base Treatment |
| 109776.73 |  |  |  |  |  |  |
| 107844.05 |  |  |  |  |  |  |
| 116005.31 | 114829.5113 | 93.31% | 1081.383 | 0.942% | 7.575% | Peroxide Treatment |
| 113877.59 |  |  |  |  |  |  |
| 114605.63 |  |  |  |  |  |  |
| 119166.25 | 118716.5324 | 96.47% | 1014.126 | 0.854% | 4.000% | 1.2 M Lux |
| 117555.32 |  |  |  |  |  |  |
| 119428.03 |  |  |  |  |  |  |
