# Supplementary material for: A systemic approach to estimate and validate RP-HPLC assay method for remdesivir and favipiravir in capsule dosage form
Source: PLoS One. 2025 Apr 15;20(4):e0321474. doi: 10.1371/journal.pone.0321474 (PMC11999136; doi:10.1371/journal.pone.0321474)
Supplement: S14 Table — (DOCX) [file pone.0321474.s014.docx]

**Table S14: System Suitability Favipiravir**

| **Area** | **% Assay** | **% Recovered** | % RSD | Mean Recovery |
| --- | --- | --- | --- | --- |
| 912364 | - | - | 0.38% | - |
| 905284 |  |  |  |  |
| 907883 |  |  |  |  |
| 913590 |  |  |  |  |
| 907883 |  |  |  |  |
| 912764 | 100.47% | 100.47% | 0.361% | 100.05% |
| 907363 | 99.87% | 99.87% |  |  |
| 906837 | 99.82% | 99.82% |  |  |
| 914352 | 100.64% | 100.64% | 0.341% | 100.40% |
| 908609 | 100.01% | 100.01% |  |  |
| 913535 | 100.55% | 100.55% |  |  |
|  | Minimum % Recovery = | |  | 100.05% |
|  | Maximum % Recovery = | |  | 100.40% |
|  | Mean % Recovery = | |  | 100.23% |
