# Supplementary material for: A systemic approach to estimate and validate RP-HPLC assay method for remdesivir and favipiravir in capsule dosage form
Source: PLoS One. 2025 Apr 15;20(4):e0321474. doi: 10.1371/journal.pone.0321474 (PMC11999136; doi:10.1371/journal.pone.0321474)
Supplement: S15 Table — (DOCX) [file pone.0321474.s015.docx]

**Table S15: System Suitability Remdesivir**

| **Area** | **% Assay** | **% Recovered** | % RSD | Mean Recovery |
| --- | --- | --- | --- | --- |
| 125696 | - | - | 0.33% | - |
| 125041 |  |  |  |  |
| 124935 |  |  |  |  |
| 125751 |  |  |  |  |
| 124935 |  |  |  |  |
| 125364 | 100.07% | 100.07% | 0.250% | 99.79% |
| 124781 | 99.61% | 99.61% |  |  |
| 124876 | 99.68% | 99.68% |  |  |
| 125631 | 100.29% | 100.29% | 0.256% | 100.07% |
| 125452 | 100.14% | 100.14% |  |  |
| 125007 | 99.79% | 99.79% |  |  |
|  | Minimum % Recovery = | |  | 99.79% |
|  | Maximum % Recovery = | |  | 100.07% |
|  | Mean % Recovery = | |  | 99.93% |
