# Supplementary material for: A systemic approach to estimate and validate RP-HPLC assay method for remdesivir and favipiravir in capsule dosage form
Source: PLoS One. 2025 Apr 15;20(4):e0321474. doi: 10.1371/journal.pone.0321474 (PMC11999136; doi:10.1371/journal.pone.0321474)
Supplement: Attachment — Submitted filename: PONE comments.docx [file pone.0321474.s016.docx]

**Reviewer Comments for Authors**

1. **Abstract**: References should not be included in the abstract.
2. **Mobile Phase Composition**: It is preferable to state the mobile phase composition in terms of ratios rather than actual volumes.
3. **Run Time Justification**: A justification is needed for the extended run time for the separation of only two drugs.
4. **Linearity Range & LOQ**: Justify the selected linearity range for both drugs. Additionally, discrepancies between the linearity range and LOQ should be clarified.
5. **Novelty Justification**: The manuscript claims novelty, but several HPLC methods have been reported in the literature for this combination. The authors should justify why their method is unique compared to existing ones (e.g., references: <https://analyticalsciencejournals.onlinelibrary.wiley.com/doi/10.1002/cem.3548?af=R>, <https://pmc.ncbi.nlm.nih.gov/articles/PMC10203959/>).
6. **Drug Purity**: The purity of the drugs used should be mentioned in the materials section.
7. **Methodology Heading (Section 2.4)**: The heading "Procedure" is vague. It should clearly define the purpose of the procedure.
8. **Target Concentration Justification**: The rationale for selecting target concentrations must be provided.
9. **Incomplete Units**: Some values in the methodology section are missing units. Ensure all numerical values have appropriate units.
10. **Precision Studies**: Percentage relative standard deviation (%RSD) should be reported for precision studies.
11. **Robustness Studies**: The robustness table should be revised for clarity, showing parameter variations and their impact more effectively.
12. **Quality by Design (QbD) Application**: The abstract mentions the application of QbD, but there is no detailed discussion on risk assessment, DOE application, MODR, or model validation in the results and discussion section. This needs to be addressed.

Overall, the manuscript lacks coherence and clarity in the presentation of results. The discussion on analytical method validation and optimization requires significant improvement to meet the standards of a high-impact journal.

**Reviewer Comments for the Editor**

After thoroughly reviewing the manuscript, I find that it does not meet the minimum scientific and technical standards for publication in **PLOS ONE**. While the study presents an HPLC method for remdesivir and favipiravir, there are major concerns:

1. **Lack of Justification for Key Parameters**: The manuscript does not justify the long run time, target concentration selection, or discrepancies in LOQ and linearity.
2. **QbD Application Not Detailed**: Although QbD is mentioned in the abstract, the manuscript does not discuss key elements like risk assessment, design of experiments (DOE), or model validation.
3. **Novelty Not Justified**: The study does not establish how this method differs significantly from previously reported methods.
4. **Formatting & Language Issues**: There are inconsistencies in terminology, missing units, and vague methodology headings.
5. **Inadequate Robustness & Precision Reporting**: The robustness table needs better organization, and precision studies should include %RSD.

Given these issues, I recommend rejecting the manuscript in its current form. If the authors significantly revise and address these concerns, it may be reconsidered for publication.
