# Supplementary material for: A systemic approach to estimate and validate RP-HPLC assay method for remdesivir and favipiravir in capsule dosage form
Source: PLoS One. 2025 Apr 15;20(4):e0321474. doi: 10.1371/journal.pone.0321474 (PMC11999136; doi:10.1371/journal.pone.0321474)
Supplement: Attachment — Submitted filename: Response to Reviewers.docx [file pone.0321474.s017.docx]

**Title of paper**: A SYSTEMIC APPROACH TO ESTIMATE AND VALIDATE RP-HPLC ASSAY METHOD FOR REMDESIVIR AND FAVIPIRAVIR IN CAPSULE DOSAGE FORM

**Manuscript reference:** PONE-D-25-03276

**Journal Name:** PLOS ONE

| **Reviewer 1.** | | | |
| --- | --- | --- | --- |
|  | **Queries** | **Responses** |  |
| 1 | In Assay method for dual drug combination by HPLC method is lacking novely and new finding. | Yes, it is a novel HPLC method in sense of composition of mobile phase and column as well as 1^st^ time coloaded in oral capsule formulation. |  |
| 2 | Chromatogram of blank, specificity, and LOQ is missing | Chromatogram of Blank, Specificity added in Specificity test, LOQ was determined from Slope of Response as per ICH guidelines. |  |
| 3 | Without any innovative steps and methods like QbD, greenness, and chemometrics, it seems simple experimental methods. For a research article, the author should incorporate new methodology, processes, and findings that add value to existing research. | Green chemistry discussed in introduction part and included in conclusion part as per instructions of reviewer. |  |
| **Reviewer 2.** | | | |
| 1 | References are cited as numbers superscripts, however are listed alphabetically, revise as per journal guidelines | Reference cited style corrected to “Vancouver” as per reviewer comments and journal guidelines. |  |
| 2 | Table no.7-degree Celsius sign missing | Corrected as instructed by the reviewer |  |
| 3 | Elaborate on significance of degradation studies in discussion | Significance of degradation studies added in 3.8 Stress degradation as per reviewer instructions. |  |
| **Reviewer 3.** | | | |
| 1 | Correct manuscript for grammatical mistakes. | Corrected as instructed by the reviewer |  |
| 2 | Write complete source information for instruments, materials and software used in the proposed research work. | Source information and software version added for customized instruments as per reviewer instructions. |  |
| 3 | Format references properly as per guidelines. | Corrected as instructed by the reviewer |  |
| 4 | The proposed method can be evaluated for greenness and whiteness profile. | Green chemistry discussed in introduction part and included in conclusion part as per instructions of reviewer. |  |
| **Reviewer 4.** | | | |
| 1 | **Abstract**: References should not be included in the abstract. | According to the reviewer instructions, references are removed from abstract. |  |
| 2 | **Mobile Phase Composition**: It is preferable to state the mobile phase composition in terms of ratios rather than actual volumes. | Corrected as instructed by the reviewer |  |
| 3 | **Run Time Justification**: A justification is needed for the extended run time for the separation of only two drugs. | The extended run time was due to remdesivir peak behavior, which shows inconsistent behavior in elution pattern. |  |
| 4 | **Linearity Range & LOQ**: Justify the selected linearity range for both drugs. Additionally, discrepancies between the linearity range and LOQ should be clarified. | Corrected as instructed by reviewer to know the response is linear (proportional to concentration), or the response may be non-linear, especially at higher concentrations. |  |
| 5 | **Novelty Justification**: The manuscript claims novelty, but several HPLC methods have been reported in the literature for this combination. The authors should justify why their method is unique compared to existing ones (e.g., references: <https://analyticalsciencejournals.onlinelibrary.wiley.com/doi/10.1002/cem.3548?af=R>, <https://pmc.ncbi.nlm.nih.gov/articles/PMC10203959/>). | Yes, it is a novel HPLC method in sense of composition of mobile phase and column as well as 1^st^ time coloaded in oral capsule formulation and described in introduction section. |  |
| 6 | **Drug Purity**: The purity of the drugs used should be mentioned in the materials section. | Drug purity has been mentioned as per instructions of reviewer in material section. |  |
| 7 | **Methodology Heading (Section 2.4)**: The heading "Procedure" is vague. It should clearly define the purpose of the procedure. | Procedure has been replaced with Experimental methodology as per instructions of reviewer. |  |
| 8 | **Target Concentration Justification**: The rationale for selecting target concentrations must be provided. | The rationale for target concentration has been describe in experimental methodology as per instructions of reviewer. |  |
| 9 | **Incomplete Units**: Some values in the methodology section are missing units. Ensure all numerical values have appropriate units. | Corrected as instructed by the reviewer |  |
| 10 | **Precision Studies**: Percentage relative standard deviation (%RSD) should be reported for precision studies. | % RSD has been corrected as per instructions of reviewer. |  |
| 11 | **Robustness Studies**: The robustness table should be revised for clarity, showing parameter variations and their impact more effectively. | Robustness studies has been elaborated for variable parameters and their impact as per instructions of reviewer. |  |
| 12 | **Quality by Design (QbD) Application**: The abstract mentions the application of QbD, but there is no detailed discussion on risk assessment, DOE application, MODR, or model validation in the results and discussion section. This needs to be addressed. | QbD has been added in results section with risk assessment, DOE and validation aspects as per reviewer instructions. |  |
| **Reviewer 5.** | | | |
| 1 | **Lack of Justification for Key Parameters**: The manuscript does not justify the long run time, target concentration selection, or discrepancies in LOQ and linearity. | The extended run time was due to remdesivir peak behavior, which shows inconsistent behavior in elution pattern. |  |
| 2 | **QbD Application Not Detailed**: Although QbD is mentioned in the abstract, the manuscript does not discuss key elements like risk assessment, design of experiments (DOE), or model validation. | QbD has been added in results section with risk assessment, DOE and validation aspects as per reviewer instructions. |  |
| 3 | **Novelty Not Justified**: The study does not establish how this method differs significantly from previously reported methods. | Yes, it is a novel HPLC method in sense of composition of mobile phase and column as well as 1^st^ time coloaded in oral capsule formulation and described in introduction section. |  |
| 4 | **Formatting & Language Issues**: There are inconsistencies in terminology, missing units, and vague methodology headings. | Terminologies changed, missing units added and vague methodology headings corrected as instructed by the reviewer |  |
| 5 | **Inadequate Robustness & Precision Reporting**: The robustness table needs better organization, and precision studies should include %RSD. | % RSD has been corrected as per instructions of reviewer in precision test. Robustness studies has been elaborated for variable parameters and their impact as per instructions of reviewer. |  |
